# How Time Pressure Matter University Faculties’ Job Stress and Well-Being? The Perspective of the Job Demand Theory

**DOI:** 10.3389/fpsyg.2022.902951

**Published:** 2022-05-26

**Authors:** Zhong Chen, Tzaichiao Lee, Xianghua Yue, Jie Wang

**Affiliations:** ^1^School of Business, Xiamen Institute of Technology, Xiamen, China; ^2^Chongqing Metropolitan College of Science and Technology, Chongqing, China; ^3^School of Economics and Management, Xiangnan University, Chenzhou, China; ^4^Guangdong University of Finance and Economics, Guangzhou, China; ^5^Stamford International University, Bangkok, Thailand

**Keywords:** job demand theory, psychological health, subjective well-being, time-related job demand, time-related self-efficacy

## Abstract

The work environment of employees has been greatly affected by the COVID-19 pandemic, and many limitations and risks can be seen until now. In addition to employees in firms, the faculty in colleges and universities also suffer from pressure and face challenges. For the purpose of performance assessment and promotion, the faculty not only needs to teach students, but also assumes the time pressure from academic research. This study discusses the process in which the faculty’s subjective well-being is affected, in an effort to learn about the job demands of the faculty under the work environment with a high level of time pressure, and the effect of these time-related job demands on their psychological health. In this study, 347 valid questionnaires were collected from universities in coastal areas of the Chinese mainland. The results show that time-related job demands have a positive impact on time pressure; time pressure has a negative impact on subjective well-being; and time-related self-efficacy can significantly mediate the relationship among time-related job demands, time pressure, and subjective well-being. On this basis, this study proposes its theoretical and practical implications.

## Introduction

In face of the increasingly grave and fickle COVID-19 pandemic, countries all over the world have to adapt their isolation policies to changes from time to time. In this context, the faculty members are facing increasingly intensified time pressure, resulting in the growing importance of discussion on time in workspace (Orlikowski and Yates, 2002). To adapt to the work pace during the pandemic, faculty members have to change their teaching and research contents (Parker et al., 2014), accompanied by the tight and fast requirements for academic work. These work-related requirements enable many faculty members to perceive the time urgency, thus bringing about negative consequences (Major et al., 2002; Chong et al., 2020; van Antwerpen and Scholtz, 2021). Since the time-related job demand is the major source of time pressure, many scholars often mix it with time pressure or directly use time pressure as the dimension related to time in job demands. Häusser et al. (2010) indicated that one of the most important job demands is time pressure, except for the workload. Demerouti et al. (2001) also presented that time pressure is one of the most important job demand dimensions, except for the physical workload and work environment. As a result, following these prior arguments (Schaufeli et al., 2008; Crawford et al., 2010), this study focuses on the time-related job demand and deems it as the important source of time pressure for faculty members to discuss the resulting consequences, i.e., job stress, and the impact of this consequence on psychological health and life of faculty members.

As defined in the job demand-resource (JD-R) theory (Demerouti et al., 2001; Bakker and Demerouti, 2007), the job demand is the primary predictor of negative consequences of job stress (van der Wal et al., 2018; Han et al., 2020; Wang and Chen, 2022). Specifically, the time-related job demand is one of the pressure sources that have the most negative effect on job attitude and well-being (Bakker and Demerouti, 2007), especially on job stress (e.g., Kleiner and Wallace, 2017; Chong et al., 2020). Job stress, as a common pressure indicator in the JD-R theory, can result in negative effects featuring emotional exhaustion, negative attitude, and loss of career self-efficacy (Maslach et al., 2001). According the view of health impairment process in the JD-R theory, job stress is not only significantly affected by job demands of different types (Llorens et al., 2006; Hakanen et al., 2008; Crawford et al., 2010; Bottiani et al., 2019), but also plays a mediating role in the relationship between job demands and outcome variables. Thus, this study aims to discuss the mediating effect of job stress, and its impact on psychological health and life of faculty members.

The time in workspace has been widely discussed in the academic community. Recent studies also highlight that the time-related job demands can bring about adverse outcomes to physical and psychological health of employees (Demerouti et al., 2001; Häusser et al., 2010). However, there is no sufficient empirical research on verifying the health loss progress from the perspective of the time in workspace, and most of such research only verify the impact of time-related job demand on job stress and job burnout, but few of them has discussed how the time-related job demands affect outcome variables of employees through job stress. Most empirical studies verifying the JD-R theory integrate job demands at multiple dimensions into a single, overall job demand (Demerouti et al., 2001; Llorens et al., 2006; Hakanen et al., 2008) and discuss a single outcome factor such as job performance. In the context of tension and anxiety resulting from the COVID-19 pandemic, the psychological health becomes particularly significant because of the direct relation between psychological attitude and behaviors. Although prior studies of psychological health factors took workers as the research object, variables are still mainly well-being (Van der Doef and Maes, 1999; Bakker and Demerouti, 2007; Xu et al., 2021), such as life satisfaction and job satisfaction. However, few studies focus on the psychological health impairment process and discuss the impact of job demands on psychological health achieved through job stress while exploring these associations. Generally speaking, no investigation has ever been conducted based on a complete psychological health impairment process, despite some studies have explored the impact of job burnout of nursing staff on their intra-role performance (e.g., Parker and Kulik, 1995; Bakker and Heuven, 2006). In consideration that psychological health is a key outcome indicator in the field of human resource management, this study aims to examine the effect of job stress in connection with time-related job demand by taking well-being and life satisfaction as an outcome variable.

Unlike the previous JD-R model (Demerouti et al., 2001; Han et al., 2020) that emphasizes job resources such as social support, job autonomy, and job feedback, Bakker et al. (2014) contended that the most significant breakthrough of the JD-R theory in recent years is its inclusion of the concept of individual resource. Individual resource is a positive self-assessment correlated with resilience. It refers to the perception of an individual against his/her capability of controlling and affecting the environment (Hobfoll et al., 2003) and emphasizes the importance of self-efficacy (Zhao et al., 2021; Cao et al., 2022). As reported by previous studies, such positive self-assessment can mitigate the impact of time-related job demands on outcomes of job stress (e.g., anxiety, depression, and job burnout), in addition to predicting goal setting, motivation, performance, job and life satisfaction, and other desired job outcomes (Judge et al., 2004; Xanthopoulou et al., 2013) helps to adapt to the work requirements and environment.

Based on the above statements, this study will make several contributions as following: (1) applying the JD-R theory to faculties in higher education institutions and discussing the effect of time-related job demand; (2) verifying the development and establishment of job stress and the relevance between job stress and subjective well-being; and (3) introducing the concept of positive psychology into JD-R model to discuss the moderating effect of time-related self-efficacy among time-related job demands, job stress, and subjective well-being.

## Literature Review

### JD-R Theoretical Model

More than 15 years have been passed since the JD-R model (Demerouti et al., 2001) was completely published. As hundreds of studies of the model were conducted, it has been applied in thousands of organizations (Bakker and Demerouti, 2017; Wang and Chen, 2022). There are too numerous retrospective and meta-analysis studies to mention one by one (e.g., Crawford et al., 2010; Nahrgang et al., 2011; Bakker et al., 2014; Bakker and Demerouti, 2017), and considerable research findings have been achieved. A majority of these research findings focus on the dual process: health impairment process and motivation process, and the interaction effect between them (Bakker and Demerouti, 2017). According to the JD-R model of early version (Demerouti et al., 2001), work environments can be discussed from two aspects: job demands and job resources, and specific job demands and resources will further be concentrated on according to the research purpose and background. Job demand means the part into which physical and mental efforts need to be continuously put, which may involve physical, psychological, social, and organization dimensions. That is why it is correlated with physical and/or psychological costs. Job resource may also involve physical, psychological, social, and organizational dimensions. Furthermore, job resource is able to (1) play a role in achieving the job-related goals, (2) reduce the job demand and the physical and mental costs resulting from job demand, and (3) motivate individuals to grow (Wang and Chen, 2022). The two job characteristics will lead to two kinds of psychological process. According to the psychological health impairment process, high-level job demands requiring continuous efforts may wear out employees’ resources, leading to the exhaustion of mental energy and psychological health problems. At the level of mental health, the well-being is taken as a key outcome variable in this study. Subjective well-being usually refers to a state in which a person is dominated by positive emotions and less pessimistic emotions occur during a certain period (Walsh et al., 2018; Peng, 2022). Some scholars argued that well-being is the subjective emotional experience from an individual, which represents people’s demands and values by virtue of real life.

In addition, as illustrated by the motivational process, the likelihood of accessing job resources will lead to organizational commitment and job engagement (Han et al., 2020). The role of job resources as intrinsic or external motivation will motivate employees to achieve their goals; employees gain a sense of satisfaction and achievement, resulting in more dedication to their work (Wang et al., 2021). The findings of some empirical studies (Hakanen et al., 2006; van der Wal et al., 2018; Bottiani et al., 2019) also verified that some job resources such as support or coaching may give rise to job engagement (Han et al., 2020). In addition to the direct impact, the JD-R model also proposes based on the interaction hypothesis that job resources can be a cushion for the relationship between job demands and negative job outcomes. Under demanding working conditions, instructors with higher temporal self-efficacy are able to deal with more job demands and the resulting stress after effect, thus mitigating negative outcomes that may originally result from high job demands (Bakker et al., 2005; Xanthopoulou et al., 2007; Bakker et al., 2010; Cao et al., 2022).

### Job Stress

In the context of the high time-related job demand, job stress may lead to emotional exhaustion of employees and the negative view on things. This will bring about tiredness, boredom, and lack of enthusiasm and sense of achievement, and throw employees into a dilemma featuring excessive impairment of resources and incapability of responding to job demands (Bakker and Demerouti, 2007). Fatigue and sense of incapability caused by job stress will reduce the available energy and subjective effort that put into work by employees (Zhao et al., 2022). As a result, employees may have no sufficient energies to maintain a good job performance (Gaillard, 2018). The intense job stress will also undermine employees’ confidence in solving job-related problems, thus producing the learned helplessness against job dilemmas (Zhao et al., 2022). If employees feel tired from the job stress, employees are able to be trapped in a vicious circle that depletes their motivations to seek for support to change their situation, leading to the reduction of job performances (Han et al., 2020).

Studies of psychological health impairment in different types of organizational environment came to a similar conclusion that the intense time pressure caused by time-related job demands would deplete employees’ physical and mental energies and cause health problems, thus adversely affecting individuals’ life and job-related well-being (Toppinen-Tanner et al., 2002; Chong et al., 2020). Demerouti et al. (2001) conducted a study of 109 German nurses. They found that the time pressure dimension in job demands can effectively predict the job stress of nurses and affect their life satisfaction. This shows that job burnout is also supposed to play a mediating role in the effect of time-related job demands on employees’ subjective well-being. Some other empirical studies also indicated that the job-related time pressure facing nurses is closely correlated with their physical and psychological impairment status and even affect their performance in attending to patients (Teng et al., 2010). Zhao et al. (2022) reported that job stress, as the source of employees’ negative cognition and psychological state, may cause poor working conditions and psychological issues, thus reducing the subjective well-being perceived by employees. To sum up, as illustrated by the health impairment process in the JD-R theory, efforts required to meet job demands can generate certain physical and/or psychological costs among employees, which will further impair their physical and psychological resources and have a negative effect on job outcomes (Bakker and Demerouti, 2007; Bottiani et al., 2019). As a result, this study assumes that time-related job demands would affect instructors’ subjective welling through the mediating effect of job stress. Thus, H1 is developed as follows:

*H1*: Job stress mediates the relationship between time-related job demand and subjective well-being.

### Time-Related Job Demand

Job burnout has been an important concern since the beginning of the 20th century, and any people who work with others, without limitation to specific races or groups, cannot escape from job burnout (Demerouti et al., 2003; Han et al., 2020; Sumner and Kinsella, 2021). Job burnout is defined as a chronic fatigue syndrome, featuring cynical conviction, negative attitude, and reduced professional efficacy in work (Maslach et al., 2001). Lee and Ashforth (1996) found in their meta-analysis research that job burnout is closely related with many types of job demands and often appears when organizations ask employees to inject too much physical and psychological resources. Crawford et al. (2010) further verified in their meta-analysis research that both challenge and hindrance demands are positively associated with job burnout. Job burnout also pays a vital role in the JD-R theory (Bakker and Demerouti, 2007). As seen from previous studies of the health impairment process “job demand-stress-job outcome” in the JD-R model, job burnout is a very common mediator for stress (e.g., Hakanen et al., 2008; Bottiani et al., 2019). When job demands are beyond an extent that is bearable, employees will be unable to work in a positive attitude, leading to emotional exhaustion and negative view on things, tiredness, boredom, and lack of enthusiasm and sense of achievement. Such status of loss in physical and psychological resources is known as the job burnout, i.e., a dilemma where resources are excessively depleted and job demands cannot be met (Bakker and Demerouti, 2007). This will further harm other physical and psychological health indicators and job attitudes. Llorens et al. (2006) conducted a cross-cultural sampling from 657 Spanish employees and 477 Dutch employees to verify the mediating role of job burnout in the health impairment process. The results showed that job burnout always significantly mediates the relationship between job demands and organizational commitment, no matter which country or occupational environment is involved, what kind of methods is used to collect information (*via* computer or paper test), and which measuring tool is used to evaluate key variables in the JD-R model. Hakanen et al. (2008) designed a panel study using the two-wave three-year follow-up data of 2,555 Finnish dentists. The results also verified the mediating role of job burnout in the health impairment process and the casual relationship among variables involved in this process. All in all, empirical studies confirmed that job burnout plays a mediating role in the health impairment process.

However, prior studies of JD-R model paid more attention to workload or emotional demand but neglected the key role of time-related indicators in measuring job demands (McCauley et al., 1994; Bakker and Demerouti, 2017). It has been early found that time is the primary source of job-related stress (Cooke et al., 2019; Huang et al., 2021) and that time-related job demands (e.g., high work pace) leave employees with less time to complete job-related tasks (Kinicki and Vecchio, 1994), resulting in impairment of physical and psychological energies, loss of sense of achievement and self-efficacy, lack of enthusiasm for work, and generation of job burnout (Bottiani et al., 2019).

Empirical studies also presented that time-related job stress will lower the subjective well-being (Huang et al., 2021). A study on 800 female employees demonstrates that there is a high probability to cause health problems, as well as intense anxiety, depression, and sense of incapability when job demands include a high work pace. Chong et al. (2020) conducted a four-week follow-up study for accountants, covering the peak working days (the end of a month) and common days. The results revealed that the time pressure caused by job demands will indeed intensify negative emotions and emotional exhaustion over time, and the two factors are exactly the primary source of job stress. Thus, H2 is developed as follows:

*H2*: Time-related job demand is positively correlated with job stress.

### Time-Related Self-Efficacy

The JD-R theory highlights the role of job resources in early time (Bakker et al., 2010), but the focus is shifted to the key role in individual resources in recent years. Besides, many studies believed that both individual resources and job resources are key factors to drive motivations and reduce stress (Bakker et al., 2005; Bakker and Demerouti, 2007, 2017). The belief and attitude deriving from such resources can be regarded as self-efficacy. The interaction hypothesis of JD-R model presents that both individual resources and job demands have an interactive effect on employees’ physical and psychological health or job outcomes (Bakker and Demerouti, 2007). There are two mechanisms. First, individual resources are supposed to mitigate the negative impact of job demands on stress because such resources can help employees cope better with job demands and reduce physical or psychological impairment. Second, the importance of individual resources will become prominent when there are higher job demands, so job demands will also enhance the positive impact of individual resources on motivations. In other words, employees with higher self-efficacy are less affected by stress perception and its consequences caused by job demands, and highly motivated to complete job tasks than those with lower self-efficacy (Zhao et al., 2021; Cao et al., 2022).

This study focuses on the time in workspace to discuss the impact of time-related job demands on job performance through job burnout. Thus, time-related individual resources are selected. Bakker and Demerouti (2007) defined individual resources as people’s belief into what extent they can control their work environment. As a result, this study argues that employees’ belief in controlling their work time is also an important individual resource, i.e., time-related self-efficacy. Time-related efficacy (Macan et al., 1990; Peeters and Rutte, 2005) refers to the extent to which employees perceive that they can control time and the self-assessment that they can arrange and complete their work before deadline (Häfner and Stock, 2010; Zhao et al., 2021).

It can be found from relevant studies that the time-related self-efficacy of employees in the job context mainly stems from the overall assessment of their own time management ability (Claessens et al., 2004). Employees with the ability to set goals and priorities will have a high degree of time-related self-efficacy. Thus, employees can respond to the job demand for fast work pace with a schedule or time plan, so that they can mitigate job burnout caused by time-related job demands without consuming too many individual resources. In addition, the time-related self-efficacy is also the self-efficacy for the work time management (Cao et al., 2022). Therefore, even in the context of high-level time-related job demands, employees still believe that they are able to meet the challenges and do a good job in scheduling and time management. Employees with high time-related self-efficacy believe that they are able to work following the time schedule and better control the work time (Häfner and Stock, 2010; Zhao et al., 2021), which helps reduce the likelihood of producing job burnout due to tight schedules. As a result, the positive effect of time-related job demands on job burnout will be weakened when employees perceive the high time-related self-efficacy. Given this, H3 is developed as follows:

*H3*: Time-related self-efficacy will negatively mediate the relationship between time-related job demands and job stress.

In face of the job demand for fast work pace, employees are more confident in manage their work time and pay more attention to time schedule and plan if they have a positive self-assessment on their time management ability. As a result, the time management efficiency will be enhanced (Häfner and Stock, 2010) to deal with the stress that may be caused by time-related job demands and lower the probability of negative outcomes. Time-related efficacy can mitigate the effect that time-related job demands exert *via* the mediating role of job burnout on job performances, or in other words, slow down the overall health impairment process (Bakker and Demerouti, 2017) caused by time-related job demands, in addition to mediating the relationship between time-related job demands and job burnout. Empirical studies also demonstrate that individual resources can significantly slow down the overall health impairment process. Based on the above statement, H4 is developed as follows:

*H4*: Time-related self-efficacy will negatively mediate the relationship between job stress and subjective well-being.

**Figure 1:**
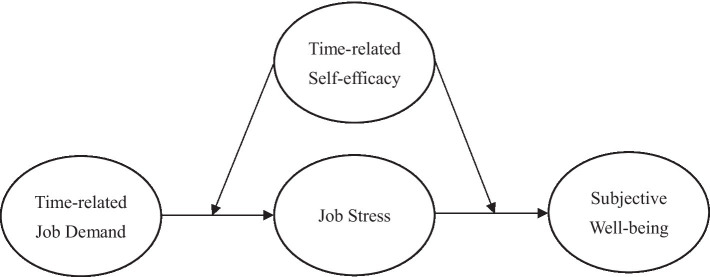
Research framework.

According to the above hypotheses, the research framework is shown in Figure 1.

## Methodology

### Sampling

In this study, data were collected by questionnaires from employees of organizations in different sectors, with a view to enhance the generalization of research findings. Researchers collected data by recruiting one supervisor and one subordinate, attempting to avoid the possibility of common method variance (Podsakoff et al., 2003). Respondents were mainly recruited from three channels: (1) reference from relatives or friends; (2) enterprises in previous industry-university cooperation project; and (3) on-the-job students in colleges and universities. Potential respondents or enterprises were first contacted *via* telephone or face-to-face interview to inform them of the research purpose and process of this study. In this study, we conducted a survey using the purposive sampling. The purposive sampling can be implemented based on the individuals’ subjective judgment and allows us to select samples that are most suitable for the purpose of the study. However, this sampling method has several disadvantages. Vulnerability to errors in judgment by researchers, low level of reliability and high level of bias, and inability to generalize research findings are three main disadvantages. To avoid these disadvantages, some conditions were set during sampling in this study, which makes the collected samples better conform to the sample reliability and to improves the generalization of the study. The results indicate that subjects do not have a significant impact on the research variables, so there is no need to include an independent variable in subsequent analyses. In the questionnaire, participants were informed of the research purpose, research ethics, and low risks, and the questionnaire information was processed in an anonymous way. After confirming their participation willingness, the paper questionnaires were posted to them or delivered to them in person, and they were also asked to hand some questionnaires over to their colleagues randomly. After completion, respondents mailed questionnaires back to researchers in sealed envelopes to further ensure the confidentiality of data. Each questionnaire is designed with numbers, which allows researchers to match questionnaires of supervisors to their subordinates after questionnaires are taken back. The unmatched questionnaires were deemed as invalid ones, and those not completed or with obviously inappropriate answers were screened out. The remaining valid questionnaires were included in the statistical analysis procedure. A total of 353 out of 500 questionnaires were withdrawn, and 347 were ultimately judged as valid ones, accounting for 70.16% of total questionnaires. The sample background is shown in Table 1.

**Table 1:** Demographic characteristics of research samples.

Variable		Size	Percentage (%)	Variable		Sample	Percentage (%)
Gender				Managerial Position			
	Male	209	60.2		Yes	142	40.9
	Female	138	39.8		No	205	59.1
Age				University type			
	30–40	135	38.9	Research orientation		63	18.2
	40–50	143	41.2	Teaching orientation		83	23.9
	50–60	59	17.0	Teaching and Research both		201	57.9
	More than 60	10	2.9	Marital Status			
Affiliate					Married	259	74.6
	Lecturer	149	42.9		Unmarried	84	24.2
	Associate Professor	183	52.7		Divorced	4	1.2
	Professor	15	4.3				

Furthermore, the study collects information from the same respondents in form of a single questionnaire, which may lead to the common method bias (CMB). In this study, the single factor verification from Harman is adopted and all the measured items are analyzed by the non-rotating matrix. The analysis results demonstrate that there are nine factors, of which the eigenvalue is greater than 1, and the explanatory variance of factor 1 is 33.23% that could not explain most of the variance. Therefore, it can be concluded from the verification results that there is no common method bias in this study.

### Instruments

Previous studies often include Karasek’s (1985) job content instrument and time-related items in their questionnaires when discussing time-related job demands or measuring time pressure in job demands, which may lead to the problem of single item (Wanous and Reichers, 1996). In order to measure time-related job demands in a more comprehensive manner, this study adopts three items for the variable “tightness orientation” in Schriber and Gutek’s (1987) work time scale. For job stress, this study adopts the scale revised by Tongchaiprasit and Ariyabuddhiphongs (2016), which owns two measuring dimensions of workload and insufficient resources, as well as 13 measuring items, such as “Lack of feedback on performance,” “Insufficient management support,” and “Poor communication between staff.” Subjective well-being adopted the scale revised by Keyes (2005), which owns three measuring dimensions of emotional, psychological, and social well-being, as well as 12 measuring items. For time-related self-efficacy, this study adopts the scale revised by Macan (1994) from the one developed by Macan et al. (1990), and there are five items in total in this scale, such as “I am clear about time required to complete different tasks” and “I think I can control my time.”

## Results

### Measurement Evaluation

The Confirmatory factor analysis (CFA) was performed to examine the internal and external reliability of the constructs. In particular, the instruments’ Cronbach alpha (α), factor loadings, composite reliability (CR), and average variance extracted (AVE) were computed. All scores obtained during the CFA were recorded above the least acceptable value, as shown in Table 2. Moreover, the satisfactory results were also computed in the case of each subgroup. Our results show the CFA of the measurement models, indicating that each construct has good convergent validity. The square root of AVE for each latent construct (see Table 2) is greater than its cross-correlation with other constructs, confirming discriminant validity. In Table 2, many correlation coefficients are lower than 0.8, although some of them are higher. This indicates that there is no multicollinearity among items in the scale.

**Table 2:** Measurement.

	1	2	3	4	5	6	7
1. TWD	0.920						
2. Workload	0.347	0.722					
3. IS	0.432	0.713	0.759				
4. Emotional	−0.249	0.013	−0.111	0.836			
5. Psychological	−0.192	0.172	0.038	0.739	0.819		
6. Social	−0.192	0.141	0.033	0.694	0.706	0.851	
7. TSE	−0.056	0.303	0.165	0.537	0.617	0.547	0.882
*α*	0.939	0.815	0.877	0.855	0.836	0.873	0.857
AVE	0.846	0.521	0.576	0.699	0.671	0.724	0.777
CR	0.956	0.867	0.905	0.903	0.891	0.913	0.913

TWD, Time-related Job Demand; IS, insufficient resources; and TSE, Time-related self-efficacy.

### Path Analysis

**Figure 2:**
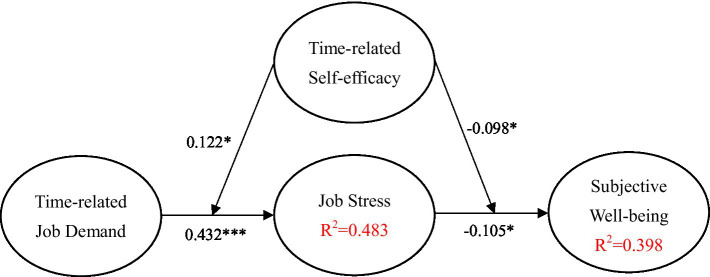
Structural model. *p < 0.05; ***p < 0.001.

After completing the confirmatory factor analysis, we conducted the path analysis and tested the structural model. The path model was tested through structural equation modeling (SEM) analysis to assess direct and indirect effects. Partial least squares structural equation modeling (PLS-SEM) was adopted to construct the structural model. Compared with CB-SEM, PLS-SEM is more suitable for this study including when the research objective is exploratory research for theory development; when the analysis is for a prediction perspective; when the structural model is complex; when the structural model includes one or more formative constructs; when the sample size is smaller due to a small population; when distribution is lack of normality; and when research requires latent variable scores for consequent analyses. The above reasons provide supports to consider the PLS is an appropriate SEM method for a study. In this study, the SRMR value was 0.048 (< 0.08) and the NFI was 0.932 (>0.90) and the dULS < bootstrapped HI 95% of dULS and dG < bootstrapped HI 95% of dG indicating the data fits the model well. Most results were significant at the *p* < 0.01 level. The empirical results are presented in Figure 2.

As indicated in the Figure 2; Table 3, job stress appeal has a negative significant effect on well-being (*β* = 0.195, *p* < 0.01); thus, H1 is supported. Moreover, time-related job demand appeal has a positively significant effect on job stress. Overall, the figure shows that job stress has a full mediating effect on the relationship between time-related job demand and subjective well-being; thus, H2 is supported. Our findings also evidence that time-related self-efficacy significantly and positively moderates the relationships of time-related job demand to job stress (*β* = 0.246, *p* < 0.001); thus, H3 is supported. Similarly, results indicate that time-related self-efficacy significantly and negatively moderates the relationships of job stress to subjective well-being; thus, H4 is supported. The Stone-Geisser Q2 values obtained through the blindfolding procedures for subjective well-being (Q2 = 0.543) and job performance (Q2 = 0.325) were larger than zero, supporting the model has predictive relevance (Hair et al., 2017).

**Table 3:** Results of hypotheses testing.

Paths	Coefficients	*t*-value	Results
H1: Time-related Job Demand → Job stress	0.432	8.303	Confirmed
H2: Job stress → Subjective Well-being	−0.105	2.221	Confirmed
H3: Time-related Job Demand*Time-related Self-efficacy → Job stress	0.122	2.322	Confirmed
H4: Job stress*Time-related Self-efficacy → Subjective Well-being	−0.098	1.985	Confirmed

**Figure 3:**
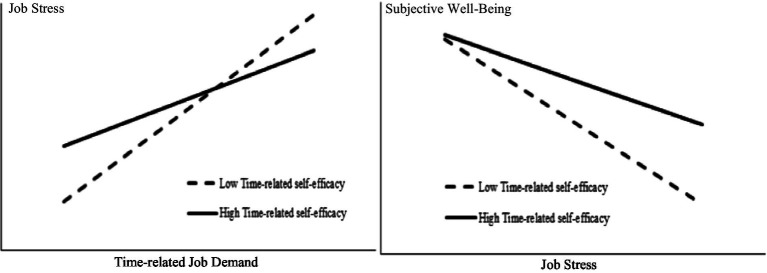
Interaction effects.

The interactions among time-related job demand, job stress, and time-related self-efficacy are significant for job stress and subjective well-being. To show the moderating effects among these relationships clearer, we plotted these significant interactions and indicated that time-related job demand and job stress significantly predicts employees’ job stress and subjective well-being only when their time-related self-efficacy is high, as shown in the simple slope chart in Figure 3.

## Conclusion

### Discussion

Unlike previous studies of JD-R model that focus on job characteristics such as workload and emotional demand in job demands, this study puts emphasis on the time-related dimensions that are seldom involved in discussions, especially for university faculty. This study adds an element, i.e., positive psychology, to the JD-R model to mediate the relationship between variables and discuss their effect on subjective well-being of the faculty from the positive cognition belief. Particularly, considering changes to job forms in the context of pandemic, the tightness orientation is designed as a main variable in this study to discuss the impact of time-related job demands on the psychological health impairment of teachers and the mediating role of time-related self-efficacy in the psychological health impairment process. The results show that time-related job demands can mediate the impairment on the subjective well-being through job stress. Furthermore, this study verifies the mediating role of teachers’ time-related self-efficacy in the relationship among time-related job demands, job stress, and subjective well-being. This study expands the research domain of JD-R model in terms of the subject of work time and offers preliminary empirical evidence.

Different from previous studies of job characteristics and job demands (e.g., Häusser et al., 2010; Bakker and Demerouti, 2017; Wang et al., 2021), this study discusses time-related demands (i.e., tightness orientation) from negative psychological features such as anxiety at work and in life in the context of pandemic. The results demonstrate that time-related job demands do have an impact on teachers’ psychological health, and thus lower their subjective well-being. It is also found that time-related job demands are significantly correlated with job stress, which is consistent with augments of other scholars. Thus, it can be seen that the strict job demands in terms of teaching and academic performance will increase the job stress of the faculty, thus intensifying their feeling of uncertainty in workplaces and affecting the effectiveness of school governance. That is, in a fast-paced and uncertain work environment, job demands will increase teachers’ job stress, covering teaching quality, academic performance, promotion demands, etc.

Given the importance and difference of varied job demands for employees’ job attitudes and performance, many scholars are attempting to clarify the efficacy of job demands in more detail. Specifically, time-related job demand is undoubtedly important, as a job demand dimension that has the greatest impact on employees in addition to workload (Häusser et al., 2010). Apart from job stress and subjective well-being verified in this study, other scholars also found that the high work pace, one of time-related job demands, also has an adverse effect on physical health. The reason may be that the high time-related demands, especially the repeated and tight work pace (Bosch et al., 2011), change the extent, frequency, and/or duration of teachers exposed to general risk factors such as the high-risk pandemic context. This is consistent with views of prior studies. Besides, it is necessary to explore the potential positive effect of job demands, and it is also worth examining in depth the potential positive effect of time-related job demands on teachers’ job attitudes and performance, and even the buffering effect of perceived stress on stress (Bakker and Demerouti, 2017). It is found from research results that the intense job stress caused by time urgency may cause the faculty has to sacrifice rest time to deal with teaching and academic work. This will lead to a low life and job satisfaction, and further reduce the subjective well-being of the faculty.

Moreover, prior studies of job resources and job control mostly highlighted the importance of the individual’s sense of subjective control over the outside world. But the research focus is often put on the degree of job control or autonomy in job characteristics, which cannot become individual resources that are applicable for all kinds of job situation and job characteristic (Hobfoll, 2002; Bakker and Demerouti, 2007). This study examines teachers’ self-efficacy on work time and contends that teachers’ perceived time control is an important individual resource. The results of this study present that time-related job demands have a significant effect on psychological health impairment and can affect the subjective well-being through job performance. On the contrary, the highly perceived time control will not only reduce the impact of time-related demands on job stress, but also lower the negative effect of job stress on subjective well-being, thus enhancing teachers’ psychological health. As in previous studies, this result also verifies the importance of time-related self-efficacy and its positive correlation with job satisfaction and subjective well-being (Claessens et al., 2004; Xu et al., 2021; Cao et al., 2022).

### Implications

Although the results verify that time pressure caused by time-related job demands will lead to psychological health impairment, the job demand for high work pace can hardly be avoided in the organizational context nowadays, especially in the context of COVID-19 pandemic. Thus, appropriate adjustment of performance evaluation, diversified communication with teachers, or enough time given teachers for preparation and planning may turn teachers’ subjective assessment of time-related job demands into a challenging stressor. Thus, this study suggests that colleges and universities should take initiatives to communicate with the faculty to obtain feedback on their self-assessment of time pressure and rebuild the subjective perception over time-related job demands through in-depth conversation with teachers, thus avoiding psychological impairment process and unnecessary negative consequences. However, in the self-statement of psychological status from students, errors may occur. Considering research ethics, if the actual psychological status of students is assessed, it may be easier to understand the connection between job demands, stress, and subjective well-being. In addition, subsequent researchers are suggested to bring interview contents and faculties observations of working status to their studies, so as to provide support for the research findings and give an overall judgment.

Prior studies have verified the relationship between time management behaviors and perceived time control. With time management behaviors, employees can perceive that they can deal with time-related conditions, increase the self-efficacy for use of work time. As a result, time management training is a good start point in order to develop the perceived time control into one of individual resources. Therefore, it is recommended in this study that managers can make good use of the important situational effect of the perceived time control on work time characteristic and its stress to increase the time-related individual resources of teachers and generate subsequent buffering and positive effects.

### Limitations

Data of different sources were used in this study to avoid the possibility of common method variance (Podsakoff et al., 2003). However, such data were collected at a single time point, making this study a cross-sectional one that has causal inferences among research variables to be clarified (Spector, 2006). The research framework of this study is based on JD-R model’s health impairment process: job demand-stress-job outcome. A variety of study designs such as longitudinal design (Hakanen et al., 2008; Bakker et al., 2014) and diary method design (Simbula, 2010) have been created to establish the casual inference of this theoretical model. Future studies are suggested to verify the health impairment process from the time perspective using the diary method or longitudinal design based on the results of this study.

The tightness orientation is considered as a major variable in this study, but there are many other orientations such as synchronization orientation and future orientation to be clarified. All these orientations can cause time pressure on employees and thus produce the health impairment process. Furthermore, in this study, employees are invited to make an overall assessment on their work time, but we suggest researchers should discuss other better time-related individual resources (e.g., multi-task capability and care for future outcomes) for specific work time characteristics.

This study is a general staff survey and is limited to the education service industry, since we expect it is applicable to the faculty in most universities. Only 347 valid questionnaires were obtained in this study. Although specific sampling conditions are proposed during sampling to ensure the sample validity, more samples will contribute to valuable insights and generalization for research results and theories. We suggest researchers expand the sampling size to reach more diversified and enriched conclusions. However, previous studies demonstrated that different work groups may have specific job demands and resources, which have a greater effect on their psychological health impairment. Therefore, different types of teachers such as senior highs school teachers or junior high school teachers can be taken into account in future studies to clarify work and/or individual resources that benefit them the most.

## Data Availability Statement

The raw data supporting the conclusions of this article will be made available by the authors, without undue reservation.

## Ethics Statement

The studies involving human participants were reviewed and approved by Academic Committee of School of Economics and Management, Xiangnan University. The patients/participants provided their written informed consent to participate in this study.

## Author Contributions

ZC and TL contributed to conception and design of the study. TL organized the database. XY performed the statistical analysis. ZC and XY wrote the first draft of the manuscript. ZC, XY, TL, and JW wrote sections of the manuscript. All authors contributed to manuscript revision, read, and approved the submitted version.

## Conflict of Interest

The authors declare that the research was conducted in the absence of any commercial or financial relationships that could be construed as a potential conflict of interest.

## Publisher’s Note

All claims expressed in this article are solely those of the authors and do not necessarily represent those of their affiliated organizations, or those of the publisher, the editors and the reviewers. Any product that may be evaluated in this article, or claim that may be made by its manufacturer, is not guaranteed or endorsed by the publisher.
